# Building up bit by bit, parent’s experiences of equine–assisted intervention among children and adolescents with mental illness: a grounded theory study

**DOI:** 10.1080/17482631.2024.2354945

**Published:** 2024-05-17

**Authors:** Ing-Marie Carlsson, Marie Bräutigam Ewe, Peter Nymberg, Henrika Jormfeldt

**Affiliations:** a School of Health and Welfare, Halmstad University, Halmstad, Sweden; b Center for Primary Health Care Research, Region Skåne: Helsingborg/Malmö, Skåne, Sweden

**Keywords:** Children and adolescents, equine-assisted intervention, grounded theory, mental health, parents

## Abstract

**Background:**

Mental ill health among children and adolescents has increased worldwide. Mental health difficulties from a young age are associated with school absence and educational underachievement. A holistic perspective of treatments besides medical treatment is essential Thus, there is a need for research regarding equine-assisted intervention (EAI).

**Purpose:**

The present study aimed to understand the outcomes of an equine-assisted intervention for children and adolescents with mental ill health from the perspectives of parents and close relatives.

**Methods:**

This study used a qualitative research design informed by Charmaz’s Grounded Theory, with a purposive sample including six in-depth interviews.

**Results:**

The theory “*building up bit by bit*” was constructed, explaining the recognition that their children/adolescents were built up bit by bit and created a stronger self-identity. The participants referred to changes in the child’s or adolescent’s way of being and emotional regulation, which constituted building blocks leading to the child’s or adolescent’s ^1.^ increased Harmony. ^2.^ enhanced Self-identity, and ^3.^ improved Capability.

**Conclusion:**

Parents and close relatives experienced that their child or adolescent was built up bit by bit and gained a stronger foundation to stand on. This led to increased harmony in everyday life with stronger self-worth, better performance, and reduced school absenteeism.

## Introduction

In Sweden and worldwide, the number of children and adolescents experiencing mental ill health is increasing, both in terms of self-experienced malaise and diagnosed mental illness (Piao et al., 2022; WHO, 2021). Along with the increased occurrence of mental ill health, psychotropic drugs are prescribed to a greater extent in both children and adolescent populations (Gómez-Lumbreras et al., 2021; National Board of Health and Welfare, 2019), often without adequate guidance for the use or dose guidelines of psychotropic drugs in children and adolescents (Gómez-Lumbreras et al., 2021; Hirsch, 2018). However, the long-term effects of psychotropic drug use on physical and psychological development among children and adolescents are not yet fully known (2007). Therefore, non-pharmacological approaches are crucial to avoid over-medicalization and institutionalization among children and adolescents with mental ill health (National Board of Health and Welfare, 2019). Thus, there is a need to develop and implement psychosocial intervention efforts to avoid long-term negative impacts on schooling and peer relationships with severe and costly social consequences (Colizzi et al., 2020; Lakhan & Hagger-Johnson, 2007). Therefore, non-pharmacological approaches are crucial to avoid over-medicalization and institutionalization among children and adolescents with mental ill health (National Board of Health and Welfare, 2019). Thus, there is a need to develop and implement psychosocial intervention efforts to avoid long-term negative impacts on schooling and peer relationships with severe and costly social consequences (Colizzi et al., 2020).

Mental ill health is interconnected with lower self-esteem, fewer social interactions, and a sedentary lifestyle with more time spent in physical inactivity (Bailey et al., 2018), affecting executive functions such as planning and performing, often resulting in poorer school results (Burnett-Zeigler et al., 2012). The negative loop of low self-esteem, social isolation, sedentary lifestyle, and stigmatization related to mental ill health negatively affects long-term behavioural development, social skills, academic outcomes, and access to the labour market later in adulthood (Bailey et al., 2018; Rock et al., 2014; Snyder, 2013; Wagner et al., 2015). Earlier research has revealed that children and adolescents experience difficulties navigating a healthcare system lacking a clear, holistic perspective and coordination of care interventions (Westberg et al., 2020). There is a need to involve children and adolescents actively in their recovery process where they perceive themselves as capable persons to avoid low self-confidence, lack of empowerment, and self-responsibility (García-Carrión et al., 2019; Xie, 2013).

Studies involving animals in the care have shown positive results regarding social interaction, communication, reduced problem behaviours, autistic severity, and stress levels among young participants with mental illness or autism spectrum disorder (Hoagwood et al., 2017; O’Haire, 2013). These interventions work particularly well among children and adolescents with difficulties verbally expressing their thoughts and feelings (Wilson et al., 2017). The internationally used umbrella term Equine Assisted Services (EAS) embraces all kinds of equine-assisted health promotion and educational interventions that incorporate horses and other equines to facilitate biopsychosocial benefits and therapeutical improvements in various health issues (Wood et al., 2021).

EAS for children and adolescents with mental ill health, including symptoms such as anxiety and depression, has resulted in improved self-esteem, self-confidence, assertiveness, life satisfaction, and self-control, as well as a reduction of problematic behaviours such as aggressiveness and assertiveness (Bachi et al., 2012; Corallo et al., 2023; Lee et al., 2016; Wilson et al., 2017). A recent interview study among children and adolescents with mental health conditions showed that the participating children and adolescents during and after they had received EAS experienced innate calmness, improved self-esteem, and self-confidence (Punzo et al., 2022). Similarly, some experimental and quasi-experimental studies have indicated that equine-assisted interventions have the potential to promote emotional and social abilities among children and adolescents with mental health problems or at risk for mental illness (Hoagwood et al., 2017). Furthermore, EAS naturally include a large amount of physical activity, which has shown the potential to reduce long-lasting psychiatric symptoms among individuals with severe psychiatric conditions (Erdner & Magnusson, 2012; Hultsjo & Jormfeldt, 2021; Nguyen Ho et al., 2023). Parents of children and adolescents with mental illness are often hampered by a higher burden of parental care and social stigma (Park & Seo, 2016), a situation which frequently negatively affects the caregiver’s (parent’s) economic situation and influence their physical, psychological, and social health (Azman et al., 2017). Previous research on relatives’ experiences of EAS among adult participants with psychotic disorders revealed that the natural milieu around the horses enabled relaxation and that the horses contributed to positive emotions and provided a healthy focus for discussion among the participants, which increased their self-confidence and motivation to participate in shared social and physical activities (Friden et al., 2022). The perspective of parents constitutes an important aspect of the outcome regarding the effects of EAS on children and adolescents with mental ill health.

## Aim

The present study aimed to understand the outcomes of an equine-assisted intervention for children and adolescents with mental health issues from the perspectives of parents and close relatives.

## Materials and methods

### Design

Since the parents’ perspective of EAS for children and adolescents with mental illness was relatively unexplored, the study adopted a constructivist grounded theory (GT) approach from research grounded in individual interviews. Grounded theory is a general inductive method that serves to learn about the actual phenomenon and is a method for developing theories or conceptual frameworks of the studied world (Charmaz, 1995).

### Sample

The sample was purposive, and the inclusion criteria were parents or close relatives of children and adolescents who participated in an equine-assisted intervention. The participants had to understand and speak Swedish or English to be included. Participants who corresponded to the inclusion criteria received oral and written information and gave their written informed consent (SFS 2003: 460) before they were included in the study. In total, six persons participated: four women and two men. All but one, who was a grandmother, were parents to the participating child or adolescent. We included one parent or one close relative to each of the children or adolescents (*N* = 6) who participated in the equine-assisted intervention.

### The intervention

The equine-assisted intervention for children and adolescents occurred at a farm in southern Sweden once a week and lasted between 60 and 120 minutes for 12 − 14 months. The children and adolescents came to the farm with a parent or another close relative who did not participate during the intervention. The sessions were led by a licenced physiotherapist certified to perform equine-assisted interventions by the National Organization for Horse-assisted Interventions (OHI). Each session included picking up the horses in the pasture, brushing and saddling the horse, and riding. Riding occurred in a separate place designed for the purpose, absent disturbing noise or other activities. Actual time spent on horseback was varied according to the participant’s needs and conditions. Each treatment session ended with the saddle off, brushing, and letting the horse back into the pasture. The horses were specially trained for this purpose and were suitable for therapeutic work. The well-being of the horses was assessed continuously at each session based on definitions of animal welfare (Fine, 2015). The participating children and youths also met dogs, cats, and hens at the farm.

When the interviews were performed, there were five girls and one boy, with the youngest participant being nine years old and the oldest 18 years old (md.11), who participated in the equine-assisted intervention. Before taking part in the intervention, all study participants had significant problems in school related to high anxiety levels, which manifested in psychosomatic pain and nausea, social phobia, compulsive obsessive disorder (OCD), and self-harming behaviour. One of the participants had been diagnosed with attention deficit hyperactivity disorder (ADHD) and had problems with impulse control and aggressive behaviour, two of the participants had been diagnosed with depression disorder, and one participant had been diagnosed with post-traumatic stress disorder (PTSD). All participants had been referred to the equine-assisted intervention by their treating psychologist or child and adolescent psychiatric clinic.

### Data collection

The data collection consisted of six open qualitative interviews with the parents or close relatives of the intervention participants, conducted by the first author (IMC), between April 26 and 27 May 2021. The introductory question was: “Can you tell me what it has meant for your child to participate in the equine-assisted intervention? Follow-up questions and in-depth questions were used. The interviews were recorded digitally and lasted between 16–51 minutes, with a median length of 41 minutes.

### Data analysis

Data were analysed through constructivist grounded theory (GT) and focused on how the participants construct meaning in relation to the area of inquiry. In GT, data collection and data analysis are carried out simultaneously (Charmaz, 2006). The interview was thoroughly listened to and transcribed. The interview was read line by line, and data was broken down into substantive codes, labelled, and written in active form by hand in the margin. When coding, we tried to use the participant´s own words, in vivo codes, so that the analysis was grounded in data and reflected their reality. Examples of initial coding were “being able to open up,” reducing volatility,” being able to focus,” and “coming to your senses.” The analysis from each interview influenced the following interview’s questions and focus. However, the interviewer tried to stay open to the emergent data. During and after the interviews, the first author made memos, a kind of written reflective thoughts, and memos were also carried out during the analysis phases.

The initial coding was followed by focused coding with concurrent data generation. In this phase, we synthesized more significant amounts of codes and constructed concepts that expressed actions and processes of what was going on when their child or adolescent took part in the intervention. These codes were compared with one another for similarities and differences. Questions such as “what, when, and how “, were used when constructing the concepts (Charmaz, 2006). One preliminary constructed concept was “being able to calm down.” However, the concept included more than just calming down. The concept described how the participants noticed that their child or adolescent during the time of intervention could calm down, get inner peace, and become more generally emotionally stable. Finally, this concept ended up as the concept of “being calm and stable”, representing one of the twelve building blocks (subcategories) that constituted the theory.

The final analysis stage was theoretical sampling, when the concept’s (major categories and subcategories) properties and relationships were saturated. At this stage, we went back and forth in the material, comparing concepts with concepts, concepts with codes, and concepts with the original transcripts. The memoing and sorting of the memos were included as data and abstract models at this stage. Theoretical sampling was employed until theoretical saturation was reached. According to grounded theory, theoretical saturation was reached in this study when no new properties in the concepts or further theoretical insights emerged to the substantive theory named “Building up bit by bit” (Charmaz, 2006).

### Ethical considerations

The Ethical Review Authority’s Operating Region Lund in Sweden, Dnr: 2019–00008, approved the study. The study conforms to the principles outlined in the Declaration of Helsinki (World Medical Association, 2013). All the participants received oral and written information about the study and its aim. They also received the information that participation was voluntary and could withdraw without explanation. The participants gave written consent before the interview was performed. The General Data Protection Regulation (GDPR) and the Swedish Act concerning the Ethical Review of Research involving Humans (SFS 2003:460, 2003) were followed to protect participants’ privacy and confidentiality of their personal information.

## Results

### The theory of building up, bit by bit

The substantive theory of *building up bit by bit* was constructed to explain how the participants recognized that their child or adolescent, during the equine-assisted intervention in small steps and slowly, over time, were built up bit by bit and gained a stronger foundation to stand on in life.

She has gained a stronger foundation to stand on. (no. 1)

**Figure 1.**
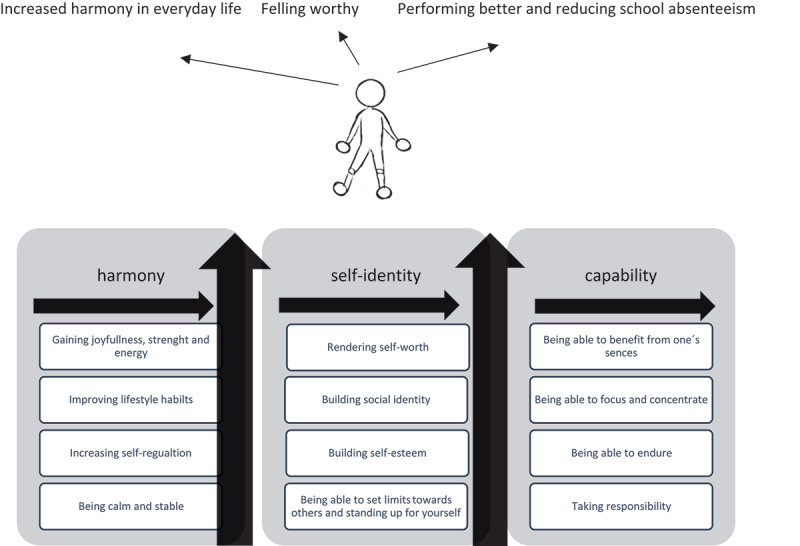
The theory of building up, bit by bit.

The participants referred to several minor changes in the child or adolescent’s way of being, behaviour, and emotional regulation, which constituted building blocks leading to the child or adolescent experiencing increased^1^ Harmony^2^ enhanced Self-identity, and^3^ improved Capability (Figure 1). Together, these changes made the child or adolescent feel enhanced harmony in everyday life, feeling worthy and performing better at school and reducing school absenteeism.

It’s incredible that we’ve come this far, even for me, because I see progress, but they are small, small steps, but a part of the journey. She knows what she wants, she didn’t know that two years ago, but now she’s starting to stack up. (no. 5)

Furthermore, the positive change for the child impacted and increased the well-being of the whole family, siblings, and parents.

It was she who was supposed to heal, but…then we were all drawn along. (no.1)

All participants stated that the equine-assisted intervention had contributed to the positive changes that had taken place. However, they acknowledged that other positive things also happened while the intervention was ongoing such as that medication had started, increased support at school, and continued support from psychologists and child psychiatrists. However, everyone was very grateful for the opportunity the equine-assisted intervention meant and concluded that the whole intervention concept with the place environment, the animal relationship, and the session’s framework was forceful and contributed to the change; not just one of these three sources was more important than the other. The participants expressed that the farm’s unique environment gave the child peace, stabilization, and growth. A comparison was made to the environment in the fairy tale of Astrid Lindgren, the Brothers Lionheart, and the Cherry Tree Valley, where the fairy tale figure gets stronger and healthier and finally rides up to the mountains with the horse. The parents also emphasized the importance of the precise framework of the intervention sessions that helped the child know what would happen during the sessions, what to expect of them, and what was allowed on the farm. Several of the children/adolescents had taken regular riding lessons before they started the intervention. However, the parents or close relatives said it was not the same. The differences mentioned were that the children/adolescents were much more involved and took greater responsibility on their own during the intervention sessions compared to their involvement in regular riding sessions.

### The three supporting building blocks building the foundation, harmony, self-identity, and capability

#### Harmony

Although the children and adolescents’ mental health issues varied, there was agreement that the intervention had improved the child’s or adolescent’s harmony in everyday life. The parents and close relatives revealed that the child or adolescent, by taking part in the intervention sessions, became more joyful, gained strength and energy, and could cope better and ability to cope with everyday life but also coping with living. All the young ones longed for the sessions every week, and taking part in the sessions created positive feelings that were transferred to other contexts, such as school.

That’s why she’s here; she needs support in meeting the horses; the horse provides peace, a completely different world. When she’s been here, she can endure another week. If she can’t be here, she has no energy to do her homework or desire to do anything. She withdraws, locks herself in her room, doesn’t even have the strength to read, which she loves, and eats only half of what she should. The horse helps her function until the next time, she eats more and thinks clearly. If it were to end, she wouldn’t cope with her life. (no.5)

Other difficulties, which improved after participating in the equine-assisted intervention were problems with self-regulation as well as behavioural and emotional regulation. Gaining balance and stabilization was emphasized in the interviewee’s descriptions of what the intervention meant for the child or adolescent.

She has learned more and more. The small exercises have had a great impact; balance in life has to do with balance in other things. (no.1)

Difficulties with mood swings and impulse control, resulting in aggressive and harsh behaviour towards others were reported in the interviews. One parent specifically reported gains in the child’s self-regulation, a more even mood, and an improved ability to control actions after the therapy sessions.

He has always been a squirrel on the move, rough towards friends. Just the joy of coming here is his own, his riding, his thing. He and I… He has changed during this year. Now there have been several changes since we moved to Sweden, an assistant at school, the medication. It’s a big difference, but it’s probably the combination, of routines, help at school, riding, medication, the mood swings have disappeared with fewer outbursts. (no.4)

Anxiety was a common problem, and an inability to regulate emotions, thoughts, and actions led to avoidance, isolation, and physical concerns. The participants experienced that the equine-assisted intervention contributed to reduced anxiety, in which the horse was important and described as a supportive and reassuring friend with whom the child or adolescent could share thoughts to reduce anxiety without risking that something conveyed in confidence was passed on to someone else.

One example of reduced anxiety and compulsive obsessive disorders was a girl with constant nausea and a fear of vomiting. Her fear ruled the whole family; everything revolved around nausea, always having vomit bags ready, and the girl could not sleep in her bed. If they would go on an excursion or invite guests, this was permanently cancelled at the last minute due to the girl’s condition, isolating them and preventing them from leaving home. The mother gave the following quotation as an example of progress and the feeling of being on the right track as the intervention sessions evolved.

She ate so poorly that she was allowed to bring her own food to school. Yes, she had a gastroscopy to rule out physical impact and took large doses of medication, but she hasn’t touched them for a year, so that’s a fantastic change… It has been very tough, so it’s nice that it’s over. Now, she rarely mentions the nausea, so I see the problems as something of the past. (no.2)

Another girl who suffered from social phobia and had not dared to ride the bus on her own for over three years managed to do so after participating in the intervention. Furthermore, the children and adolescents’ lifestyle habits improved, sleep deprivation was reduced, and appetite increased.

#### Self-identity

The participants expressed that the children’s and adolescents’ self-identity were changed positively after participating in the equine-assisted intervention. For example, when the equine-assisted intervention started, all five girls were described as shy. Shy of being in large groups and reserved towards other persons, some girls had social anxiety and withdrew from publicity.

…she is very shy and still is, but she has become much stronger. (no.3)

She was shy at the beginning and felt insecure. There is a big difference now; she is much better. (no.6)

Moreover, the girls were also described by the participating parent or close relative as being afraid of many things, like not wanting to sleep over with friends, being afraid of animals, being afraid of commitments and making mistakes, and being unfavourable evaluated. The girls were also fearing what was going on in the world. Being shy and afraid created uncertainty and feelings of fear of being inferior to others, which affected their view of themselves and their relation to others. However, participating in the intervention meant that the children and adolescents were allowed to be who they were, which made them feel confirmed and less afraid.

She has been allowed to have opinions, feel like herself here, and it has made her less flighty and less afraid. (no. 1)

According to the participants, the equine-assisted intervention affected their child or adolescent and made them open their minds, become more aware of who they were, feel proud and satisfied with being at the farm, and let peers know what they did there, which rendered a stature of self-worth and social identity.

She has gained a different self-confidence; she has felt inferior … she feels proud in front of her friends at home, “this is what I do’”. (no. 3)

Just the joy of coming here. This is his own, this is his riding, his thing. He and I, we come here every week. (no. 4)

In turn, self-confidence increased, and the participants noticed that the child or adolescent’s belief and trust in themselves and their abilities increased as the therapy sessions evolved.

She has grown, self-confidence, self-esteem. ‘I can handle this’. She doesn’t turn away from the uncomfortable like before. (no. 1)

All the participants described that they noticed increased self-esteem in their children or adolescents and that they appreciated and valued themselves more after participating in the equine-assisted intervention. Comments such as “she had no self-esteem before” or “extremely low self-esteem” were mentioned, and that a change for the better had occurred. The horse was referred to as contributing to building up the child’s or adolescent’s self-esteem bit by bit. This made the child or adolescent stand up for themselves, and what matters to them and set reasonable limits in relation to others.

She has dared to assert herself a bit more. She has always been cautious; now, she has toughened up a bit more and can talk about things that have happened at school and how she has stood up for herself. Before, it was about fitting in and not standing out. Today, she dresses the way she wants; she dares to and can say, ‘I don’t care, Mom’, she has grown so much. (no. 2)

#### Capability

The equine-assisted sessions at the farm were described as a way for children and adolescents to be present at the moment, right there, taking a break, which enabled them to calm down and come to their senses.

It becomes a breathing pause during the week they are just here and now; I think it is precious. (no. 6)

The participants considered this breathing space necessary for their children or adolescents since they all had problems with concentration and focus at the start of the intervention. Some children were described as” *being all over the place, totally confused, restless or volatile*.” However, the intervention sessions improved these problems.

The place, with animals and guidance and talking with a therapist, meant a different possibility for children with concentration difficulties and sitting still, compared to ordinary care from a psychologist or psychiatrist.

When she started coming here, she was unfocused. She looked at you when you talked to her but heard nothing. When she got instructions, she didn’t remember. She became very calm here, and she was afraid to assert herself, afraid to make mistakes, cautious, confused, very confused, and agitated in all contexts. Her gaze was very distant; she wanted to but was very closed. Now, there is a difference; at school, she has started to perform; at home, she is calmer, understands when you talk to her, doesn’t nag, isn’t flighty, and has no blockage that was there from the beginning, and is more content with herself, she longs for this every time… She was restless, didn’t want to talk, just flee, flee; there was never a stop, she needed to fill something… here she is calm… the therapist calms her down. (no. 1)

Absolutely, it has been worth it compared to going to an office; then, he would have lost concentration immediately. Then I think it’s much better to be with the animals. There are no gray areas here; she’s very much like this, and this is how it works (the therapist). I appreciate that; I like straight talk, no coddling. (no. 4)

The children’s and adolescents’ difficulties in concentration and focus and various disorders such as anxiety, mood disorders, compulsive obsessive disorders, and lack of impulse control resulted in poor school attendance. Some did not have enough energy to attend school, and others had difficulties attending school or school-related activities, staying at school, or did not feel well at school. This changed for the better as they attended the equine-assisted intervention, and they could gradually concentrate and endure the whole day at school during the intervention sessions.

It’s a completely different girl… It took a toll before; she only went to school until lunch, but she has hardly had any absences at all now in 8th grade. She manages and looks forward to it and talks about sleeping over with friends. School absenteeism has decreased; she is happier and more open now. The obsessive thoughts have decreased, and she sleeps better. (no. 6)

She has gained a different sense of calmness; she has always had difficulty concentrating on her homework… She has also realized what it has done for her. Before, she was at home at least once a week from school, and she got anxiety attacks because of it. We almost never see that now; it’s been over six months since it happened. (no. 2)

Moreover, the child or adolescent grows as a person during the equine-assisted intervention, by respecting the animals, and other people and by learning to take responsibility for themselves and their commitments safe understandable, and manageable environment. This developmental process was successively built up by the session’s framework where the child or adolescents first took care of the horse, then started riding, and finally, after several sessions, took care of and responsibility for a specific horse.

## Discussion

The results show that the participants in this study experienced positive developmental changes within their children or adolescents when the equine-assisted intervention was added to previously ongoing supportive interventions such as medication, increased support at school, and continued support from psychologists and child psychiatrists. The equine-assisted intervention does not at all focus on symptoms, diagnosis, or medical treatment but instead offers a context with loving and caring relationships in a playful encouraging context with a focus on the child’s or adolescent’s resources in accordance with individual preferences. The experiences of the young study participants themselves have previously been presented (Punzo et al., 2022), showing their perspective of the equine-assisted therapy intervention as an indispensable prerequisite for their recovery towards personal development by providing the peace, security, and empowerment they needed but had not been able to find elsewhere. Previous investigations reveal that young people often don’t get the support they need from the traditional healthcare system due to a lack of coordinated care interventions from a holistic perspective considering the child’s or adolescent’s entire life situation (Westberg et al., 2020) Furthermore, children and adolescents experiences of being labelled and dominated by healthcare professionals has been described as a significant barrier for involvement in treatment and often misunderstood as the severity of mental illness by healthcare professionals (Bjonness et al., 2020). It is considered plausible that the equine-assisted intervention provided an opportunity for the children to counterweigh their experiences of stigmatization, pacification, and hopelessness that traditional psychiatric care often entails and thus constituted a required precondition for their positive development.

In the present study, the interviewed participants who were a parent or a close relative witnessed that the equine-assisted intervention enhanced mental health among their participating children or adolescents and indirectly also affected the whole family by contributing to a reduced care burden, social stigma, and exclusion related to mental ill health. This finding is supported by Azman et al. (2017) and Park and Seo (2016), who described that the positive changes in the children’s and adolescents’ well-being also positively affected the parents’ and relatives’ well-being, indicating that the perspective of parents constitutes an important outcome aspect of EAS for children and adolescents with mental ill health.

After the equine-assisted intervention in the present study, the participants described that they had noticed decreased school absence among the children or adolescents. Preceding research has indicated negative school results due to mental illness (Burnett-Zeigler et al., 2012) with adverse long-term effects on academic outcomes (Bailey et al., 2018; Wagner et al., 2015). EAS has previously been shown to facilitate empowerment processes and enhance self-confidence, self-esteem, and ability to handle responsibility (Xie, 2013) and reduce problematic behaviours such as aggressiveness and assertiveness (Bachi et al., 2012; Corallo et al., 2023; Lee et al., 2016; Wilson et al., 2017). EAS can help children and adolescents overcome the adverse effects of mental illness and thus facilitate participation in activities to support their experience of being a capable person (García-Carrión et al., 2019). The decreased school absence in the present study may positively affect long-term academic outcomes among the young EAS participants. Accordingly, EAS aligns with Agenda 2030 (United Nations, 2015) goal number three and goal number eleven by promoting sustainable health and well-being among children and adolescents, improving both physical and mental health, and contributing to sustainable communities by providing access to safe and green public places for children and adolescents regardless of their parents’ financial situation.

### Methodological considerations

The strengths of using the grounded theory method are that the theory of building up bit by bit and its related concepts are all grounded in data. The words used by the study participants from the results increase the study’s rigour, giving a true reflection of the reality of the interviewee (Chiovitti & Piran, 2003). The fact that only six participants were included could be considered a weakness in the study. Low (2019), who had critically discussed the concept of theoretical saturation, concluded that the richness of data in a small sample size may be sufficient for saturation and that there is no specific number of participants to determine saturation. However, the interviews in this study were empirical, original, and in-depth, with a median length of 41 minutes, giving rich data. This rich data, together with the methodological use of constant comparison going back and forth in data, comparing parts of the contents, and by using memos, was the adequacy of data to develop the properties of the categories and the theory until no new properties emerged and ensure theoretical saturation, i.e., conceptual rigour (Charmaz, 2006). Credibility was enhanced by following the method’s guidelines, and explicit transparency in the method section enhanced the credibility, further strengthened by including verbatim quotations (Charmaz, 2014). This study demonstrates quite positive outcomes for children and adolescents with mental ill health who took part in an EAS intervention. Our findings are in line with other EAS studies with participants who had autism spectrum disorders (Xiao et al., 2023), attention-deficit/hyperactivity disorder, or cerebral palsy (Ahn et al., 2021). Therefore, it could be argued that the findings are transferable to different contexts and children or adolescents with different health problems.

## Conclusion and implications

Parents and close relatives experienced that their child or adolescent was built up bit by bit and gained a stronger foundation to stand on in life. The substantive theory of building up bit by bit was constructed to explain how the parents or close relatives recognized that their child or adolescent, during the equine-assisted intervention in small steps and slowly, over time, were built up bit by bit and created a foundation to stand up for themselves. This led to increased harmony in everyday life with stronger self-worth, better performance, and reduced school absenteeism.

The originality and usefulness of the study is that it offers new and essential insights into the impacts of equine-assisted interventions for children with mental health issues, such as anxiety and school absenteeism, from the perspectives of parents and close relatives. These insights are important aspects of EAS outcomes and useful in the education and clinical work of physicians, psychiatrists, psychologists, nurses, physiotherapists, and occupational therapists. Continued research needs to be focused on replicating the results in larger studies and on how each profession may integrate EAS into their professional practice to offer children and adolescents with mental illness care and support based on a holistic health-promoting perspective.
